# Machinability of Zirconia Toughened Mica Glass Ceramics for Dental Restorations

**DOI:** 10.14295/bds.2021.v24i1.2114

**Published:** 2020-12-22

**Authors:** Sivaranjani Gali, Suresh R. Chiru

**Affiliations:** 1 Department of Prosthodontics, Faculty of Dental Sciences, M.S. Ramaiah University of Applied Sciences, Bangalore; 2 Department of Mechanical Engineering, Faculty of Engineering & Technology, M.S.Ramaiah University of Applied Sciences, Bangalore

**Keywords:** Machinability, Dental Ceramics, Mica Glass-Ceramics, Dental Zirconia, Tool penetration rates, Usinabilidade, Cerâmica dentária, Vitrocerâmica de mica, Zircônia dentária, Taxas de penetração de ferramentas

## Abstract

**Objective:**

For a dental material to be machinable for CAD/CAM technology, it must offer convenient machining, under a given set of cutting conditions. Quantitative evaluation of machinability has been assessed in literature through various parameters such as tool wear, penetration rates, surface roughness, cutting force and power. A machinable ceramic will typically demonstrate a higher tool penetration rate with signs of reduced diamond tool wear and edge chipping. The purpose of this in vitro study was to evaluate the feasibility of machining an experimental ceramic, 20 wt.% zirconia reinforced mica glass ceramics (G20Z) for indirect dental restorations and compare the tool penetration rates of G20Z to commercially available dental ceramics, Presintered Zirconia (PSZ) and IPS emax CAD.

**Material and Methods:**

Precursors of base glass (SiO_2_ -Al_2_O_3_ -K_2_O -MgO-B_2_O_3_ -F) were melted at 15000C for 2 h in a platinum crucible and quenched in deionised water. The glass frit was ball milled with 20 wt. % YSZ (G20Z) and subject to two stage heat treatment in a muffle furnace. Specimens of G20Z (12 × 2 mm) were evaluated for their feasibility of machining under varying spindle speed, depth of cut, and feed rates. Influence of depth of cut, spindle speed and feed rate (vc=8000-16000 rpm, d=0.4-0.8 mm, f=0.1-0.3 mm/tooth) on cutting forces, material response, surface roughness and tool wear were investigated. Tool penetration rates, tool wear and margin chipping were also evaluated and compared with Pre-sintered Zirconia (PSZ) and e.max CAD in a custom dental milling surveyor at 30,000 rpm with a load of 0.98 N under water lubrication for 6 min. Tool penetration rates were calculated as the ratio of length of cut and milling time with a measuring microscope and scanning electron microscope was used for tool wear and edge chipping. ANOVA and Tukey Kramer tests were used for statistically comparing the means of each group.

**Results:**

Spindle speed and feed rate play a significant role in influencing surface roughness, thrust force, cutting forces and tool wear. Penetration rates of G20Z (0.32 ±0.12 mm/min) was significantly greater than PSZ (0.26 ±0.06 mm/min) and IPS e.max CAD (0.21 ±0.05 mm/min). SEM observations reveal tool abrasion and edge chipping regardless of the ceramic type.

**Conclusion:**

High spindle speeds delivers low cutting forces with an average surface roughness of 1.61 *µ*m, with abrasive wear of the tool insert and brittle fracture of zirconia mica glass ceramic composites. G20Z with its machinable nature demonstrates greater tool penetration rates than PSZ and IPS e.max CAD. Tool wear and edge chipping is seen in all the investigated ceramics.

## Resumo

**Objetivo:**

Para que um material odontológico seja usinável para a tecnologia CAD / CAM, ele deve oferecer uma usinagem conveniente, sob um determinado conjunto de condições de corte. A avaliação quantitativa da usinabilidade tem sido avaliada na literatura por meio de vários parâmetros, como desgaste da ferramenta, taxas de penetração, rugosidade da superfície, força de corte e potência. Uma cerâmica usinável normalmente demonstrará uma maior taxa de penetração da ferramenta com sinais de desgaste reduzido da ferramenta de diamante e lascamento da borda. O objetivo deste estudo in vitro foi avaliar a viabilidade da usinagem de uma cerâmica experimental, 20% em peso de cerâmica de vidro de mica reforçada com zircônia (G20Z) para restaurações dentárias indiretas e comparar as taxas de penetração da ferramenta de G20Z com as cerâmicas dentais comercialmente disponíveis, Zircônia Presinterizada (PSZ) e IPS emax CAD

**Material e Métodos:**

Precursores de vidro base (SiO_2_-Al_2_O_3_ -K_2_O -MgO-B_2_O_3_ -F) foram fundidos a 15000C por 2 h em um cadinho de platina e temperados em água deionizada. A frita de vidro foi moída com 20% em peso de YSZ (G20Z) e submetida a tratamento térmico em duas fases em mufla. Amostras de G20Z (12 × 2 mm) foram avaliadas quanto à sua viabilidade de usinagem sob variação de velocidade do fuso, profundidade de corte e taxas de avanço. A influência da profundidade de corte, velocidade do fuso e taxa de avanço (vc = 8000-16000 rpm, d = 0,4-0,8 mm, f = 0,1-0,3 mm / dente) nas forças de corte, resposta do material, rugosidade da superfície e desgaste da ferramenta foram investigadas. As taxas de penetração da ferramenta, o desgaste da ferramenta e o lascamento da margem também foram avaliados e comparados com Zircônia pré-sinterizada (PSZ) e e.max CAD em um topógrafo de fresamento dentário personalizado a 30.000 rpm com uma carga de 0,98 N de lubrificação subaquática por 6 min. As taxas de penetração da ferramenta foram calculadas como a razão entre o comprimento de corte e o tempo de fresamento com um microscópio de medição e um microscópio eletrônico de varredura foi usado para o desgaste da ferramenta e lascamento da borda. Os testes ANOVA e Tukey Kramer foram usados para comparar estatisticamente as médias de cada grupo.

**Resultados:**

a velocidade do fuso e a taxa de avanço desempenham um papel significativo em influenciar a rugosidade da superfície, força de impulso, forças de corte e desgaste da ferramenta. As taxas de penetração de G20Z (0,32 ± 0,12 mm / min) foram significativamente maiores do que PSZ (0,26 ± 0,06 mm / min) e IPS e.max CAD (0,21 ± 0,05 mm / min). As observações do SEM revelam a abrasão da ferramenta e o lascamento da borda, independentemente do tipo de cerâmica.

**Conclusão:**

As altas velocidades do fuso proporcionam baixas forças de corte com uma rugosidade superficial média de 1,61 *µ*m, com desgaste abrasivo do inserto da ferramenta e fratura frágil de compósitos de cerâmica de vidro de zircônia. G20Z com sua natureza usinável demonstra maiores taxas de penetração da ferramenta do que PSZ e IPS e.max CAD. O desgaste da ferramenta e o lascamento da borda são vistos em todas as cerâmicas investigadas.

## Introduction

Technological developments such as Computer Aided Design (CAD) and Computer Aided Machining (CAM) have made significant impact to the field of dentistry, particularly in the fabrication of dental restorations. A dental CAD/CAM system typically consists of a digital scanner, data processing software with a manufacturing technology. The system generally uses power driven Computer Numerically Controlled (CNC) machines with advanced cutting tools to shape the preferred dental material to a desired geometry [1]. Dental materials used for CAD/CAM technology range from feldspathic ceramics to mica, leucite, lithium disilicate based glass ceramics and the polycrystalline oxide ceramics such as zirconia and alumina. Methods of hard and soft milling have been used for dental glass ceramics and pre-sintered or green zirconia blanks respectively [2]. Some of the popular commercially available dental CAD CAM ceramics are the IPS e.max CAD (Ivoclar Prosthetic System) and Pre-milled Zirconia (Katana, IPS ZirCAD,3M Lava). IPS e.max CAD is available in a pre-crystallised state (lithium meta silicate) to facilitate ease of machining and requiring further sintering to garner strength with lithium disilicate.[3]

Dental CAD CAM systems have the prime advantage of milling dense homogenous ceramic blanks with reduced flaws and have expectations of faster milling, greater material removal rate, reduced tool wear with smooth crown margins [4]. However, the current challenges of CAD CAM system include ensuring good quality of margins, minimizing digitization errors, surface modeling errors and improving the milling tool accuracy [5]. The existing CAD CAM dental materials have certain limitations such as the apparent low strength of glass ceramics for load bearing restorations and the lack of adhesive bonding of zirconia, its associated low temperature degradation and veneer fractures [6–15]. Considering the troubles of additional crystallisation of lithium disilicate glass ceramics for machining and the Archard wear equation prediction of increased possible tool wear while machining zirconia, it is important to explore for alternate machinable ceramic combinations [4,16–19].

Mica containing glass ceramics (SiO_2_-Al_2_O_3_-MgO-K_2_O-B_2_O_3_-F) can be considered as alternate restorative materials due to their machinability, bioactivity and resemblance to tooth colour [20–22]. Their classic house of cards microstructure of phyllosilicates and the nature of weak basal planes between the silicate chains, explains the machinable nature of mica glass ceramics [23]. In our preliminary studies, 20 wt.% of zirconia reinforced mica glass ceramic composites have demonstrated a brittleness index of 2.5 *µ*m ^1/2^, with Vickers hardness of 9.2 GPa and fracture toughness of 3.6 MPa/m^1/2^. With moderate mechanical properties of mica glass ceramics, zirconia’s contributing effect on enhancing fracture toughness of mica glass ceramics has been investigated by the authors [24]. With the limitations of the current dental ceramics and a desire for high strength and esthetic ceramics, novel and alternate material combinations must be explored with a goal of improving the success rate of all ceramic restorations. An evaluation of feasibility of machining an experimental ceramic, 20 wt. % zirconia reinforced mica glass ceramics (abbreviated as G20Z throughout the manuscript) for possible application as dental restorations was performed.

For a dental material to be machinable for CAD/CAM technology, it must offer convenient machining, under a given set of cutting conditions. The material must offer a reasonable surface finish, abrasiveness, an acceptable tool life with low power consumption [25,26]. In a typical in-office chairside milling set up, the software program instructs the path of the machine tool at a given feed rate and spindle speed resulting in variable cutting forces of the tool on the material. Generally, high feed rate delivering low torque or spindle speed is essential for easy machining [27,,4]. Quantitative evaluation of machinability has been assessed through various parameters such as tool wear, surface roughness, cutting force and power. Tool wear is an important criteria for machinability, as it directly affects the tool’s surface integrity and the associated machining cost. In general, a longer life of the machining tool demands high tool hardness with minimal wear. Microstructure, hardness and surface quality of the work piece substrate can determine the performance of the cutting tool [28,29]. Surface roughness of the substrate, can be related to the tool set up and its associated properties [29]. Cutting force of the tool, provides an approximation of the power requirements and estimates its impact on a tool’s endurance [29]. Machinability of dental ceramics, has also been influenced by their mechanical properties, other than the cutting tools [30]. To be machinable, the material demands a balance in the hardness (resistance to cutting) and toughness (resistance to failure) of the material. Machinability can be estimated by the parameter ‘brittleness index’, defined as the ratio of hardness to fracture toughness of a material [31]. A brittleness index of less than 4.3 *µ*m^-1/2^ is usually required for a material to be machinable [32]. Studies on machining dental ceramics have been conducted with dental hand-pieces and diamond burs under certain loads. [33–35] Rotary diamond tools have been used at the chair side clinic, for dental ceramic prosthesis adjustment. Studies on the effect of diamond cutting tools on the micro-damage of dental ceramics were investigated [36–41]. Specific cutting energy rates, cutting speed, material removal rates, diamond tool wear, chipping, machining forces and surface roughness were evaluated for predicting machinability of dental ceramics to determine the material’s restorative application and restorative quality. [33–35,42–45]

A machinable material will demonstrate greater material removal rate or tool penetration rate, low cutting forces and cutting energy with signs of reduced tool wear and edge chipping [46]. Tool penetration rate (mm/min) at a given force and cutting speed have been used to assess machinability of CAD CAM dental ceramics (4). The objectives of the present study are to:

1. Evaluate the feasibility of milling mica glass ceramic composites and investigate the influence of depth of cut, spindle speed and feed rate on cutting forces and surface roughness of the ceramic workpiece substrate with a polycrystalline diamond (PCD) tool.
2. Evaluate and compare the tool penetration rates and edge chipping to the various CAD CAM dental ceramics (G20Z, IPS e.max CAD, Pre-sintered Zirconia) at a specific cutting speed and investigate its machining influence on the cutting tool and the dental ceramics. The null hypothesis of the study was that there will be no difference between the tool penetration rates of the investigated machinable dental ceramics.

## 2 Materials & Methodology

### 2.1 Glass preparation & heat treatment schedule of G20Z

The predecessors of base glass composition (47.2 SiO_2_ -16.7 Al_2_O_3_ -9.5 K_2_O -14.5 MgO-8.5 B_2_O_3_ -6.3 F were melted at 15000C for 2 h in a platinum crucible and subsequently quenched in deionised water. The powdered glass frit was ball milled with 3 mol % YSZ (20 wt. % YSZ) (D_50_≈ 50nm) (TOSOH, Japan). A two stage heat treatment sequence of 800°C at a heating rate of 25°C/min and 1080°C with a slower heating rate of 10°C/min for 48 h was followed to densify the green glass ceramic-ZrO_2_ powder compacts in a muffle furnace (Carbolite, UK). The samples will be addressed as G20Z throughout the paper. Mechanical, wear and optical characterization with cytocompatibility and chemical solubility has been previously published by the authors [47,48].

### 2.2 Specimen preparation

Discs (12 × 2 mm) of sintered mica glass ceramic composites (G20Z) were fabricated and hand polished with #600 to #2500 SiC papers, 0.5-1*µ* diamond paste and ultrasonicated. Control specimens (12 × 2 mm) of commercially available CAD CAM dental ceramics (IPS e.max CAD and Pre-sintered Zirconia (Katana) abbreviated as PSZ, were further cleaned and ultrasonicated.

### 2.3 Feasibility of machining zirconia toughened mica glass ceramic composite (G20Z)

Rectangular specimen (30×30×26mm) of sintered mica glass ceramic composites was fabricated and polished with #600 to #2500 SiC papers and 0.5-1*µ* diamond paste and ultrasonicated. Two fluted polycrystalline diamond coated tool insert (SECOMAX, PICO end mill, India) was used to mill the mica glass ceramic composite workpiece. Computer numerically controlled (CNC) milling machine was employed to conduct the experiments, with a spindle power of 22 KW and a maximum spindle speed of 25,000 rpm for 20 min. During machining, thrust, radial and cutting forces (torque) were measured with a milling tool dynamometer, connected to charged amplifiers and a personal computer. Acquisition software was used to convert the values to digital data. The influence of different levels of the cutting parameters on thrust force, feed force, cutting force (torque) and surface roughness were investigated in the study. Different levels of cutting parameters such as spindle speed, depth of cut and feed rate and the output results are listed in Table I.

The rationale for selecting the levels of the machining parameters such as the spindle speed, feed rate and depth of cut mentioned in Table I, was to exercise caution towards tool life, and to avoid any possible damage to both the tool and the ceramic workpiece material, G20Z. Tool topography and material response after machining were examined with scanning electron microscope (SEM) to identify their surface morphology and wear mechanisms. Surface roughness values were measured immediately after the turning process at five different locations on work piece by using surface roughness tester. The surface roughness tester (Surf test SJ-210, Mitutotyo) was used to measure the surface roughness values (R_a_) of machined samples with a cut-off length of 0.25 mm and probe speed of 5mm/sec. The average of five roughness values was taken as an arithmetic surface roughness (R_a_). Analysis of influence of the various parameters was done through statistical technique. The Taguchi method based orthogonal array (L_9_) was selected for reducing the number of experiments and to minimize the effects of the factors out of control.

### 2.3 Apparatus for Machinability test

**Figure 1:**
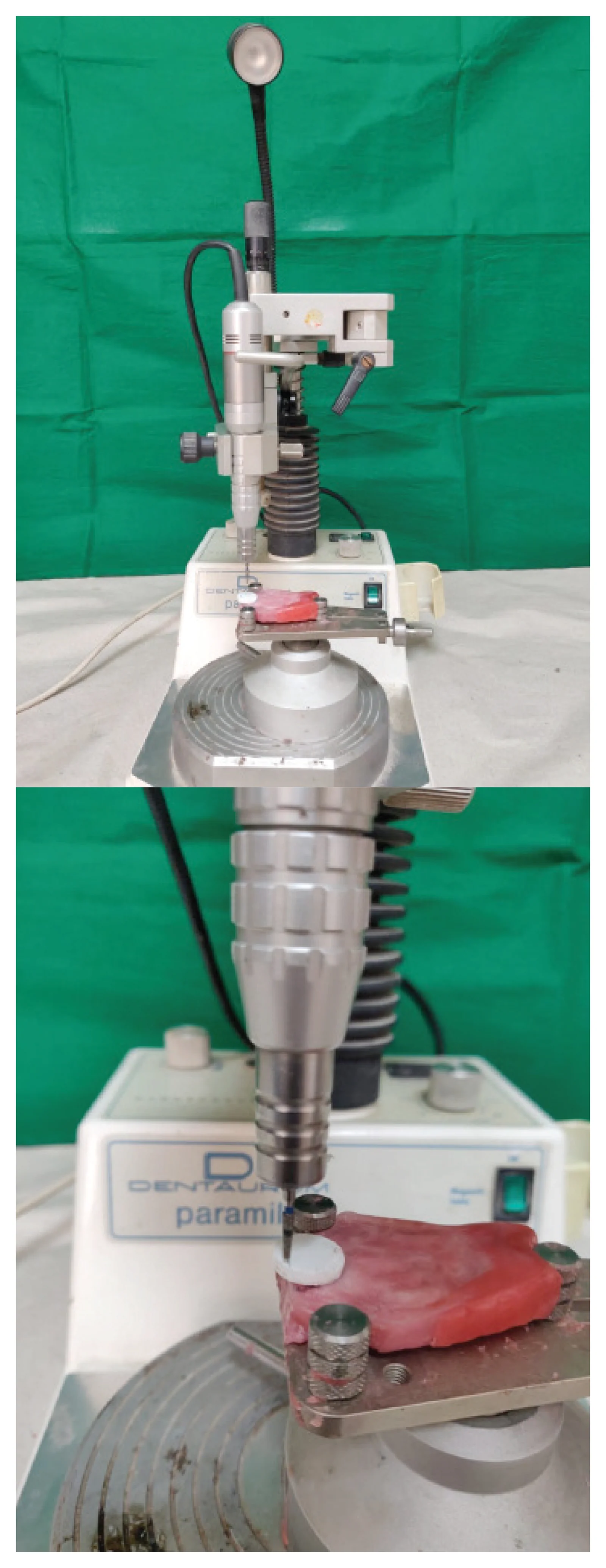
(a) Dental milling surveyor (b) Ceramic specimen on the table contacting the milling tool attached to the handpiece.

Milling was performed on a dental milling surveyor (Dentaurum, Paramil 3) to evaluate the feasibility of machining the experimental mica glass ceramic composite (G20Z) and compare to the commercially available IPS CAD and Pre-sintered Zirconia. A dental milling surveyor was customized due to technical difficulties in adapting a Computer Numerical Controlled (CNC) machine for custom milling as shown in Figure1a and 1b. Dental milling surveyors are typically used for milling guide planes, rest seats and intracoronal restorations in cast partial dentures. They are essentially equipped with a hand-piece that can accommodate cutting tools, a spindle and a movable model table. The surveyor has features of control unit and a hand-piece providing variable speed count up to 30,000 rpm with a torque of 3 Ncm. In order to closely mimic the radial cutting depth of the cutting tool in a typical CAD CAM set up, the diamond tool was made to contact towards the edge of the ceramic specimen during milling at 30,000 rpm. The tool set-up (4mm of the tool immediately above its tip contacts the specimen and a 100-g free weight attached to the hand piece of the surveyor for a constant force of 1 N), the time for milling (6 min) were based on Chavali’s paper. New diamond burs (DIA-BURS, TF -11, MANI, INC, Japan) of 106-125 *µ*m grit size (standard, blue coded, 1.4 mm head diameter, 19 mm length) were used to mill each of the experimental mica glass ceramic composites (G20Z) and the commercially available CAD CAM dental ceramics. Necessary lubrication was provided by water spray at 300 ml/min. Ceramic specimens were mounted and secured with auto-polymerizing acrylic resin (DPI-RR, Cold Cure) on the model table of the surveyor.

### 2.4 Evaluation of Tool penetration rates and Edge chipping

Tool penetration rates were evaluated for quantifying machinability amongst the dental ceramics. Thirty six depths of cut on each of the ceramic groups (n= 12) were measured using measuring microscope (Olympus STM7-CB) and tool penetration rates were calculated as the ratio of the length of cut to the milling time. An average of three readings of the cutting depths was taken. Further, tool wear and edge chipping of the dental ceramic specimens were qualitatively evaluated with electron microscopy. Ceramic specimens were gold coated and examined with scanning electron microscope (COX I EM-30 AX, South Korea) using the secondary electron (SE) mode at 20 kV and spot size of 5 nm for tool topography and edge chipping.

### 2.3 Statistical Analysis

Mean and standard deviation of tool penetration rates were calculated. One way Analysis of Variance (ANOVA) followed by Post hoc Analysis was used to compare the means of penetration rates of each ceramic specimen using SPSS Windows (Version 22.0, 2013). The level of significance [P-Value] was set at P<0.05.

## 3 Results

### 3.1 Feasibility of machining G20Z

Effect of parameters such as spindle speed, feed rate and depth of cut on thrust force, feed force and cutting forces during machining zirconia mica glass ceramic composites have been evaluated in the present study. Table I demonstrates the three factor levels for spindle speed, depth of cut and feed rate. It can be observed the values of thrust force, feed force and cutting force are lowest at spindle speed of 16000 rpm, at a depth of cut of 0.6 mm and a feed rate of 0.1mm/tooth. The results in Table I reveal a trend of low cutting forces with high spindle speeds, with varying depth of cut and feed rate. Table I also depicts the effect of spindle speed, depth of cut and feed rate on surface roughness. Surface roughness indicates surface integrity of the workpiece material and therefore, a smooth surface finish of the workpiece is expected, particularly for dental ceramics.

ANOVA results in Tables II show statistically significant influence of spindle speed on thrust force, feed force and cutting forces (p= 0.002), (p= 0.006), (p= 0.003) respectively with feed rate. However, depth of cut or tool penetration does not influence cutting force or surface roughness. As shown in Table II, feed rate also has significantly influenced surface roughness of the ceramic substrate.

The correlation between cutting parameters and output responses like, cutting forces and surface roughness are determined from regression equations (Eq-1-4), as mentioned below,

**Thrust force (Ft)**, N= 152.056 - 0.00933333 Spindle speed (s) in rpm + 112.5

Depth of cut (d) in mm + 175 Feed per tooth (f) in mm/tooth

(Eq.1) $$\begin{array}{c} \text{S}=16.2392,\text{R}-\text{Sq}=90.94\text{\%},\text{R}-\text{Sq}(\text{adj})=85.51\text{\%}).............................. \end{array}$$

**Feed force (Ff)**, N = 222.056 - 0.018875 Spindle speed (s) in rpm + 181.667

Depth of cut (d) in mm + 186.667 Feed per tooth (f) in mm/tooth

(Eq.2) $$\begin{array}{c} \text{S}=40.8804,\text{R}-\text{Sq}=84.10\text{\%},\text{R}-\text{Sq}(\text{adj})=80.57\text{\%}).............................. \end{array}$$

**Cutting Force (Fc)**, N = 264.667 - 0.0161667 Spindle speed (s) in rpm + 110

Depth of cut (d) in mm + 133.333 Feed per tooth (f) in mm/tooth

(Eq.3) $$\begin{array}{c} \text{S}=29.4573,\text{R}-\text{Sq}=87.01\text{\%},\text{R}-\text{Sq}(\text{adj})=82.22\text{\%}).............................. \end{array}$$

**Surface roughness (Ra)**, Microns = 4.48389 - 0.000193333 Spindle speed (s) in rpm + 0.375 depth of cut (d) in mm + 3.35 Feed per tooth (f) in mm/tooth

(Eq.4) $$\begin{array}{cc} \text{S}=0.464063, & \text{R}-\text{Sq}=89.96\text{\%}\\ \text{R}-\text{Sq(adj)}= & 87.93\text{\%}).............................. \end{array}$$

**Figure 2:**
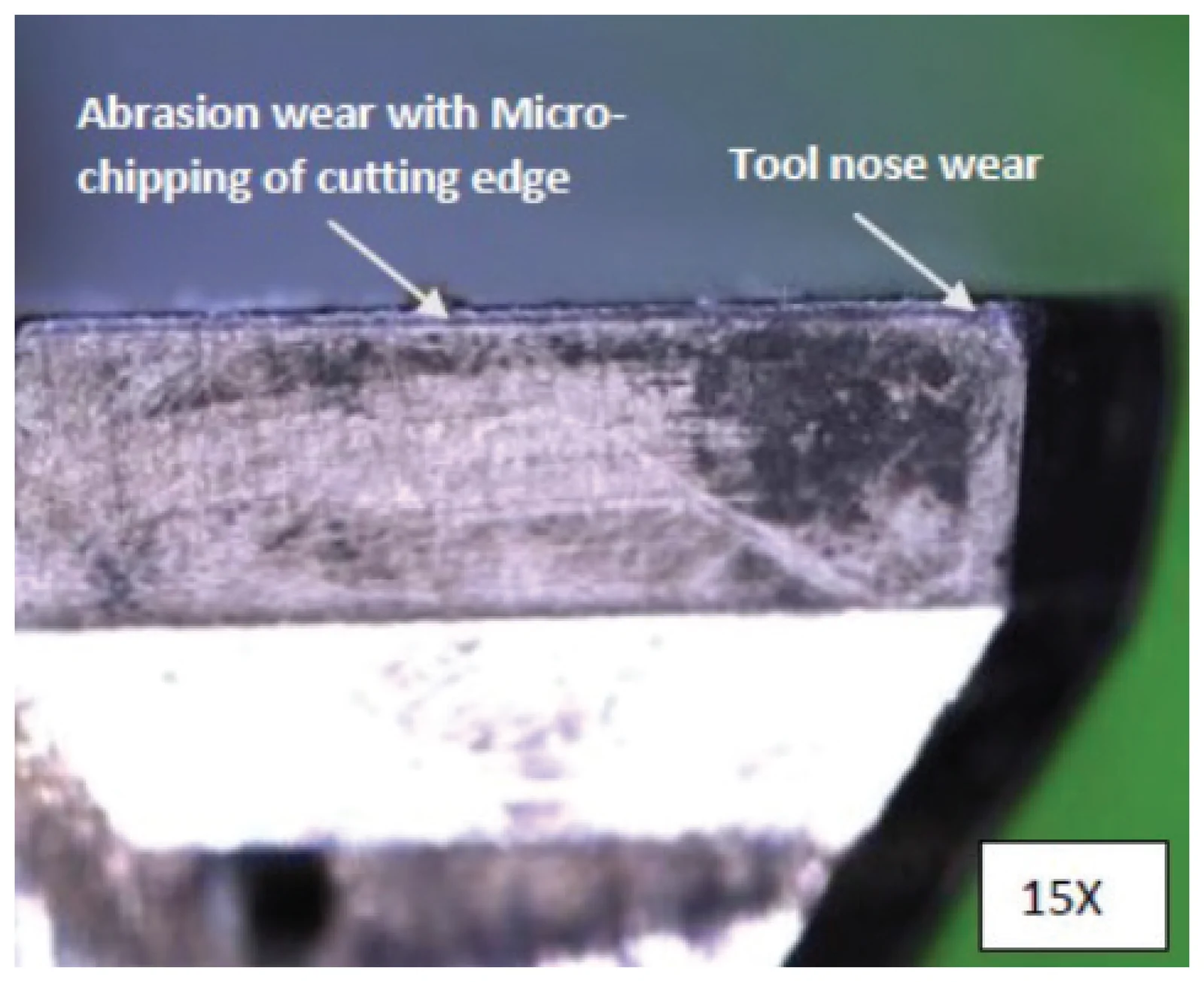
Microscopic image (15X) of worn edges of PCD tool insert demonstrating chipping of cutting edge and wear at the nose at vc=16,000 rpm, d=0.6 mm and f= 0.1mm/tooth

**Figure 3:**
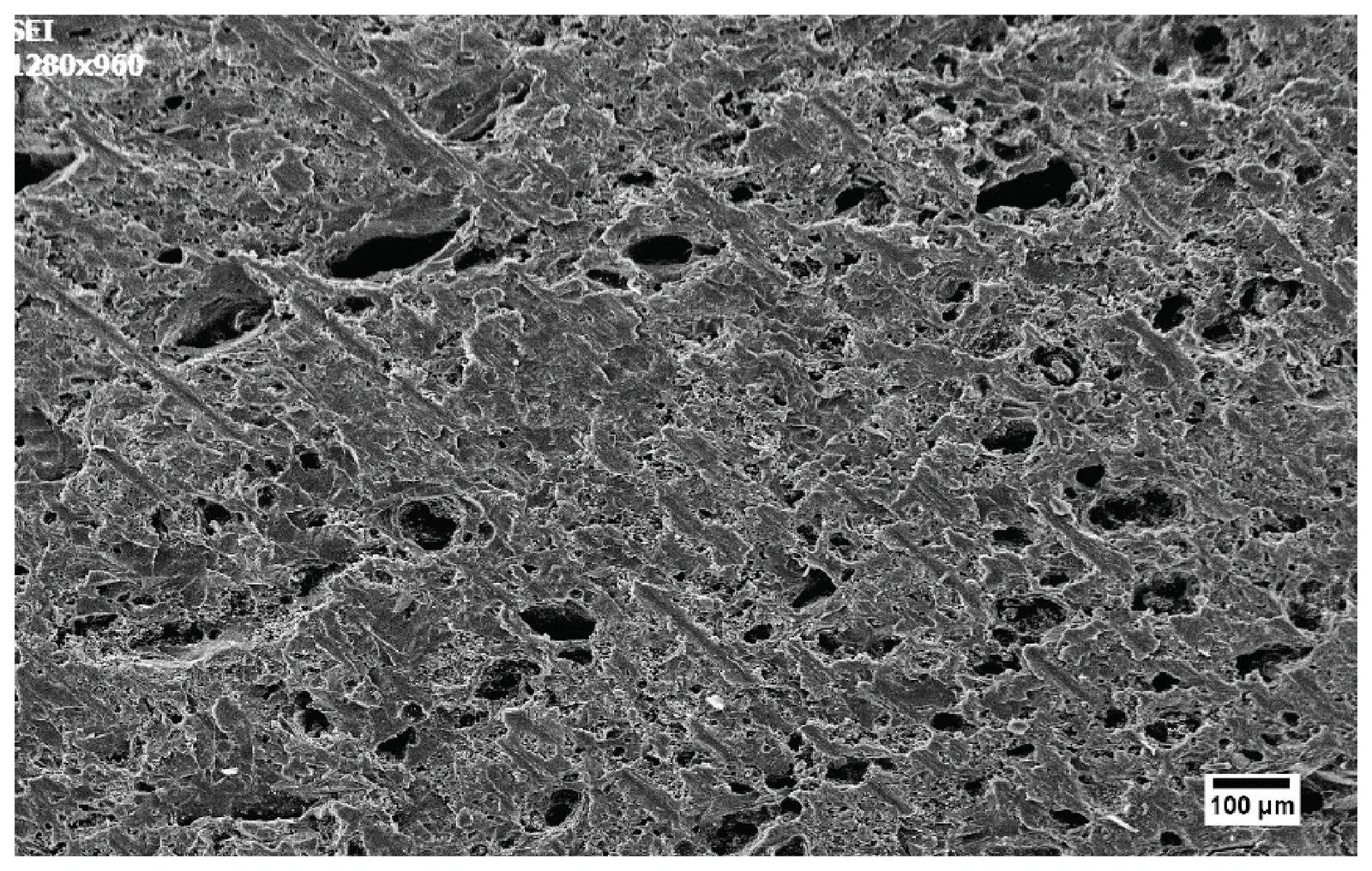
SEM micrograph (100X) showing PCD tool tracks with ground furrows on the experimental zirconia mica glass ceramic (G20Z)

**Figure 4:**
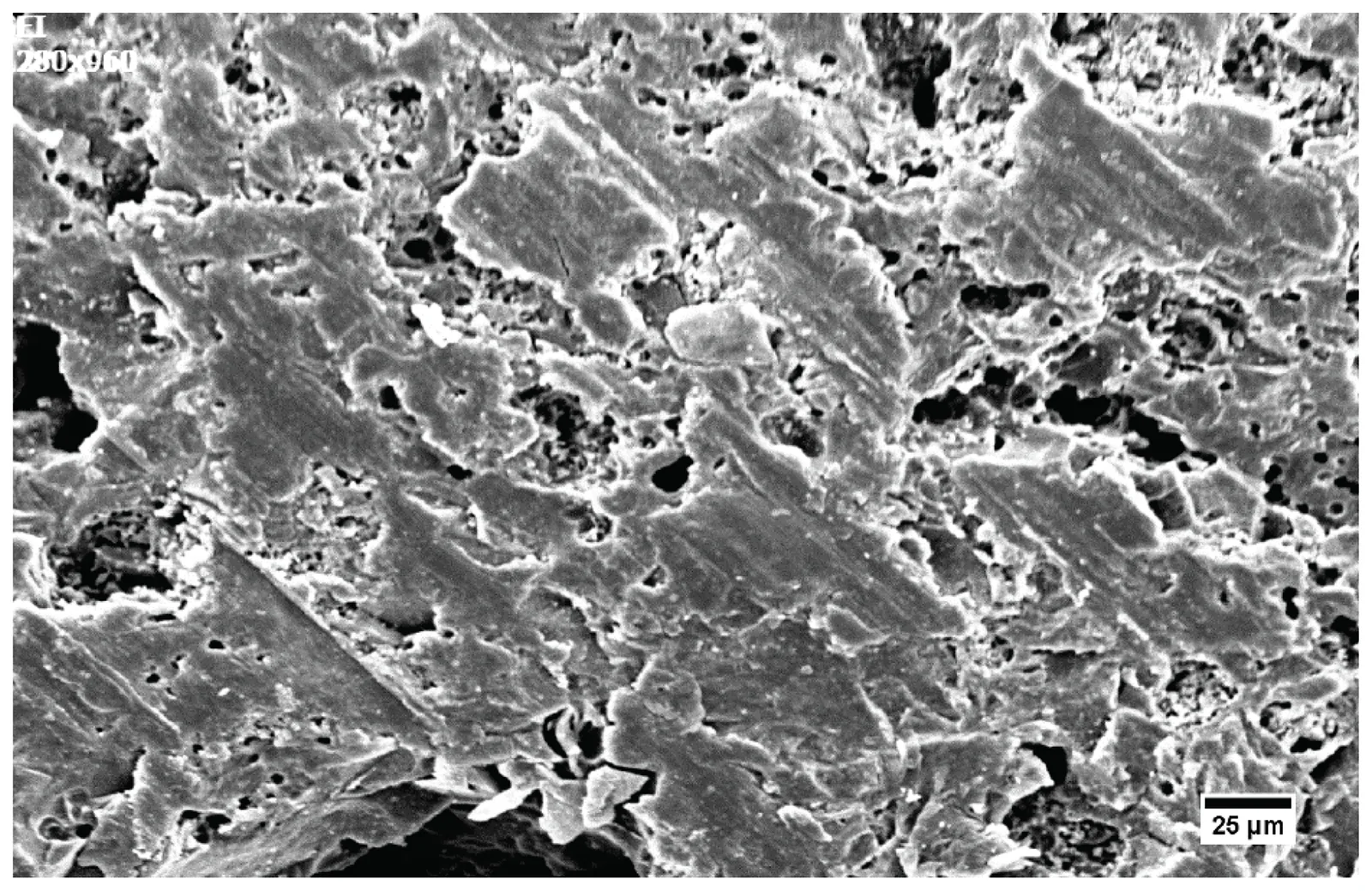
SEM image (500X) of the machined zirconia mica glass ceramic (G20Z) showing brittle fracture and trans-granular chipping (vc=16,000 rpm, d=0.6 mm and f= 0.1mm/tooth)

**Figure 5:**
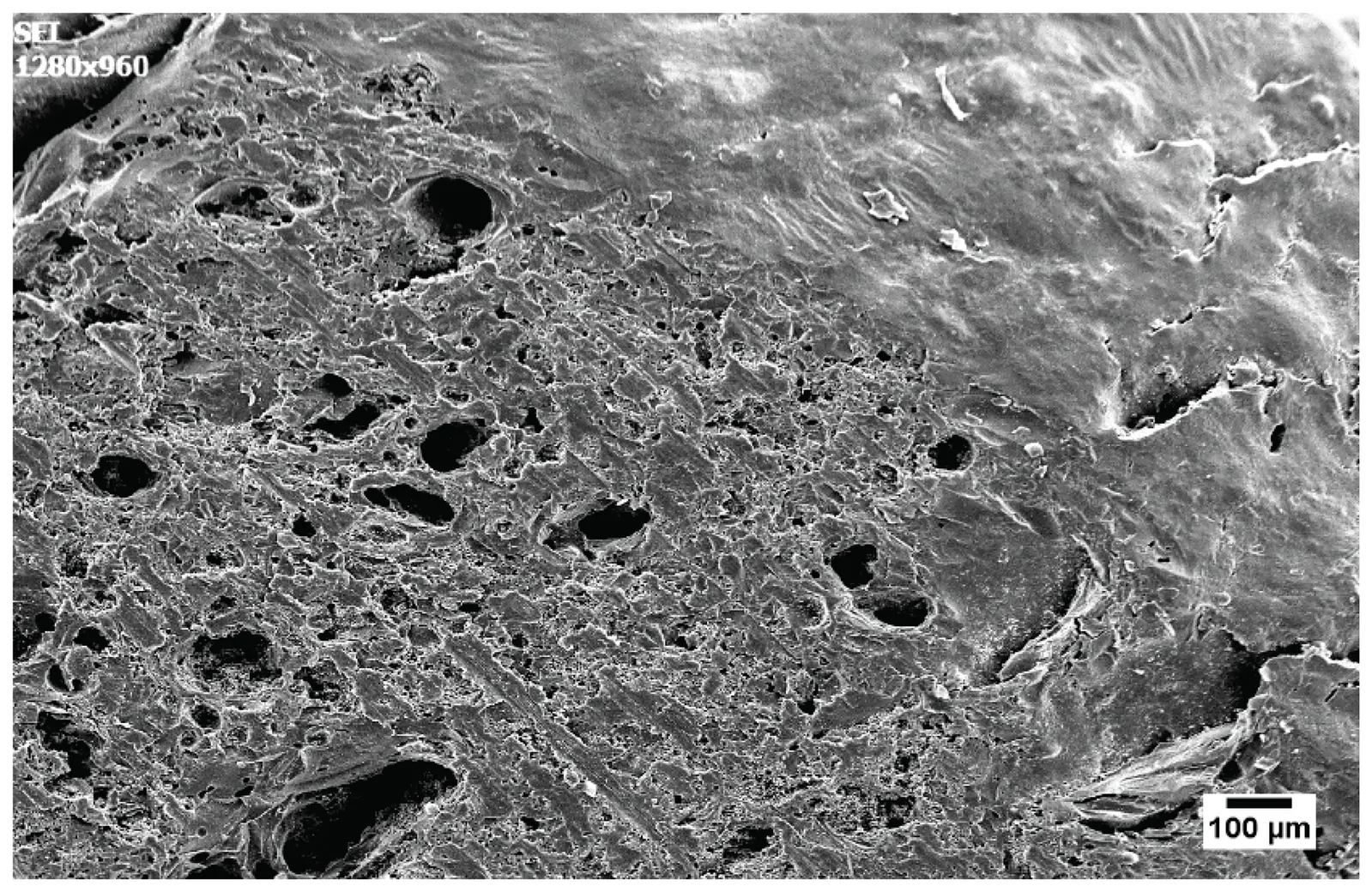
SEM image (300X) showing distinct boundaries of machined and polished surface of G20Z (vc=16,000 rpm, d=0.6 mm and f= 0.1mm/tooth)

The performance of the PCD tool has been studied in relation to cutting forces, surface roughness and tool wear. Figure 2 shows the micrographic image of the progressive wear of the PCD tool, demonstrating an abrasion wear mechanism with micro-chipping of entire cutting edge of the tool. It can be interpreted that increase in tool wear occurs with increase in cutting speed, feed rate and depth of cut. SEM images of G20Z at a spindle speed of 16000 rpm, 0.6 mm depth of cut and 0.1 mm/tooth of feed rate, under low magnification (100X) demonstrate tool tracks on the workpiece, visible as ploughing striations seen in Figure 3, and under higher magnification (500X), fractured areas, chipping with micro-fractures can be deciphered in Figure 4. Distinct boundaries of machined and polished surface of the experimental ceramic can be seen in Figure 5 under magnification (300X).

### 3.2 Tool penetration rates and Edge Chipping

Table III demonstrates mean and standard deviations of the tool penetration rates of investigated dental ceramics. It can be seen from Table III, that the differences in the tool penetration rates between the groups was statistically significant (p= 0.03).

Multiple comparison of mean differences between different groups in Table IV, reveals IPS CAD group showed significantly lesser mean tool penetration rate as compared to G20Z group at p= 0.02. The IPS CAD also showed relatively lesser tool penetration rate as compared to Pre-Sintered Zirconia (PSZ) and similarly PSZ showed relatively lesser tool penetration rate as compared to G20Z. However, the differences between IPS CAD and PSZ; PSZ and G20Z was not statistically significant at p= 0.48 and p= 0.21, respectively. This infers that the mean tool penetration rate was highest in G20Z group followed by PSZ and least in IPS CAD group.

**Figure 6:**
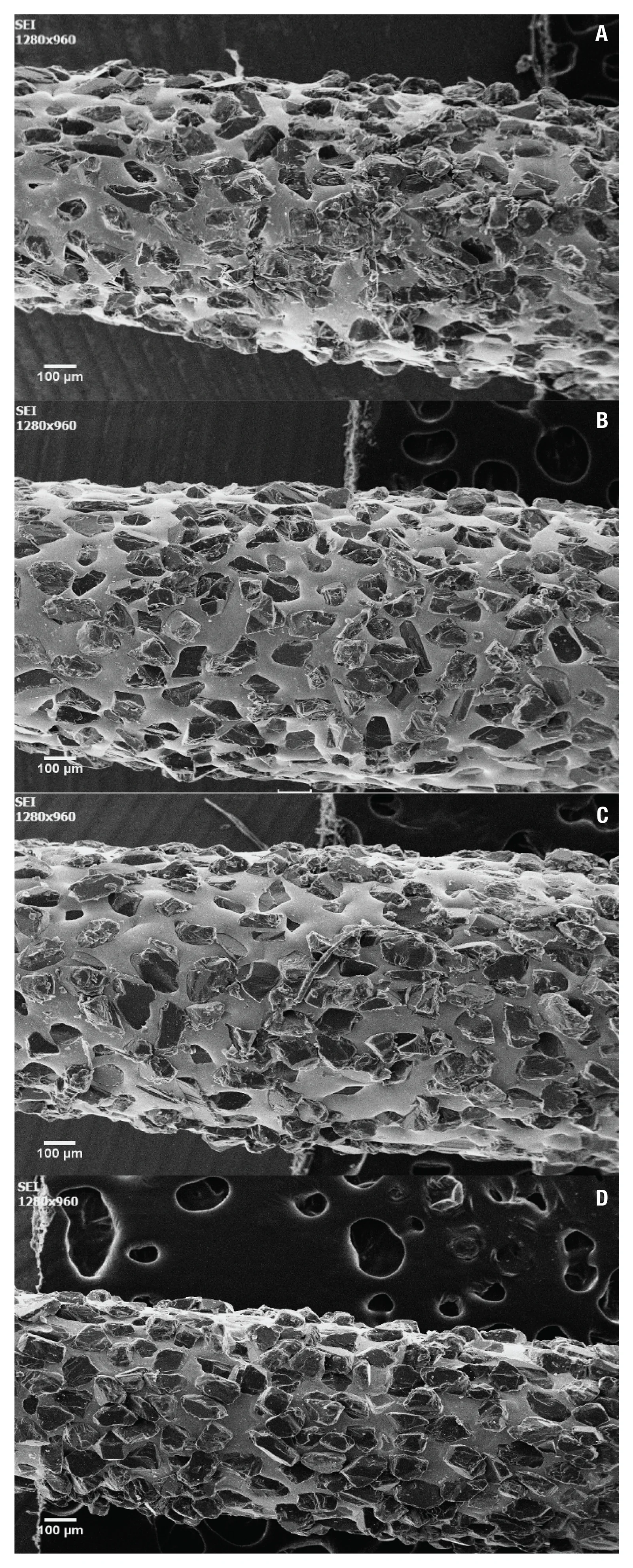
Scanning electron microscopic (SEM) images of milling tools used for (a) G20Z (b) Pre-sintered Zirconia (PSZ) (c) IPS e.max CAD (d) Control (Original Magnification X 100.)

**Figure 7:**
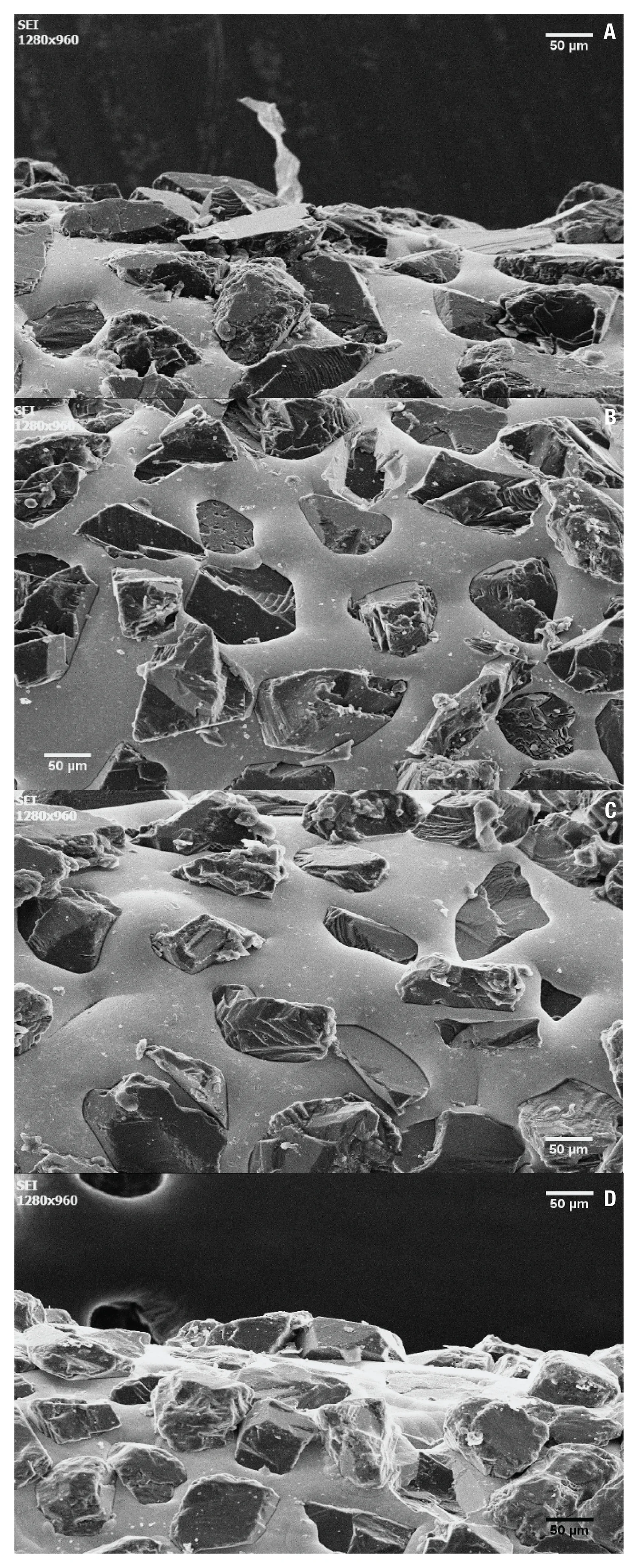
Scanning electron microscopic images of milling tool used for (a) G20Z (b) PSZ (c) IPS e.max CAD (d) Control (Original magnification X300.)

**Figure 8:**
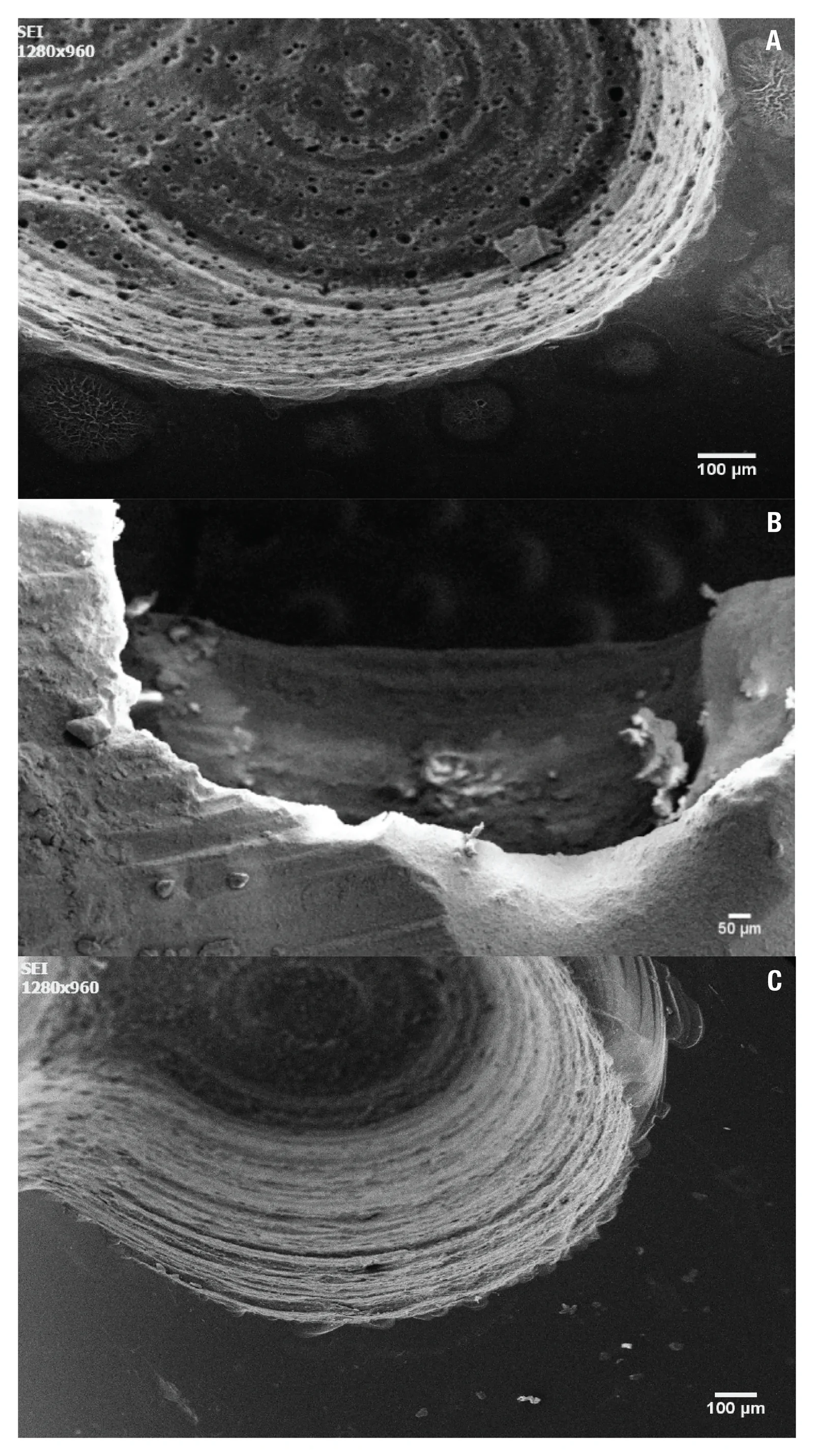
Images of specimens cut with milling tool (a) G20Z (b) PSZ (c) IPS e.max CAD.

Diamond particle wear of the milling tools while milling G20Z, PSZ and IPS e.max CAD are evident in Figure 6a-c. In contrast, the apparent absence of wear or abrasion on the control (unused diamond tool) can be perceived in Figure 6D. Higher magnifications of the diamond tool further reveals chipping of diamond particles in all the ceramic specimens (Figure 7a-c) and the control tool showing absence of diamond chipping in Figure 7d. The SEM images of the penetration depths on the ceramic specimens reveal edge chipping in all the ceramic specimens in Figure 8a-c. The edges of G20Z show margin chipping with inherent porosities visible along its cutting surface in Figure 8a. PSZ appears with irregular edge chipping on its surface and IPS e.max CAD presents with edge chipping with smooth cutting surface in Figure 8b-c.

## Discussion

### 4.1 Feasibility of machining G20Z

Estimation of cutting forces is important as they affect heat generation, tool wear and quality of surface finish of the work piece material [18]. The cutting forces that develop during machining generally depend on the origin of force such as cutting, gravitational, frictional, centrifugal forces or inertia. Such forces can be further characterized into tangential, radial, axial components, to further understand their influence. It is important to recognize, such resultant forces can affect energy consumption, wear of the tool, generation of cutting zone temperature, substrate surface quality, noise and mechanical threshold of the tools [49].

Ideally, a combination of low resultant machining forces with a smooth surface finish of the substrates are favorable for machining process. A rough finish of the substrate can pose risks of subsurface damage and catastrophic fractures. It can be observed that high cutting speeds reduce surface roughness. The reasons could be related to high cutting speed, leading to faster removal of the substrate. However, depth of cut does not seem to influence surface roughness of the substrate. Feasibility of machining a fully sintered zirconia was done using diamond tool insert. A mixed full experimental factorial design was used to study the influence of depth of cut, spindle speed and feed rate on tool wear and surface finish of zirconia. Surface roughness below 0.6*µ*m, Ra was obtained, independent of the cutting parameters with a brittle ductile material removal mechanism [50]. Similar response models of cutting parameters such as tool life, surface roughness and cutting force were evaluated for turning Inconel 718 nickel based alloy with coated and uncoated carbide inserts [26]. Surface roughness and cutting forces with mathematical models were performed on AISI 4340 steel using multi-layer coated carbide tool [29,50]. Specific cutting energy, micro-hardness, particle size determination and difference in bending strength before and after drilling can be reasonable parameters for evaluating machinability of fluormica glass ceramics [35,51,52].

In the present study, surface roughness of zirconia mica composites demonstrates its dependency on spindle speed and characteristic brittle fracture material removal mechanism. A smooth finish of a dental ceramic restoration prevents plaque accumulation, its associated periodontal problems and enhances the material durability. Generally, a diamond polished and a glazed dental porcelain exhibits surface roughness (Ra) in the range of 0.12-0.15 *µ*m respectively [53]. The resultant roughness (1.61 *µ*m) of the glass ceramic composite could be related to its surface porosity and low density associated with pressureless sintering. However, with advanced sintering techniques, the glass ceramic composites can be milled with ease at high cutting speeds with smooth surface finish. Thus, an analysis of such resultant forces during machining was done to understand their nature, estimate cutting power requirement and possibly propose new designs of cutting tools for chairside milling.

Signs of edge chipping of the tool can be seen at the entrance as the tool contacts the workpiece and impacts the workpiece. The edge chipping could be due to the hardness of zirconia particles in the mica glass ceramic composite as it strikes the tool edge during machining process. The apparent high hardness of the ceramic substrate with abrasion tool wear could lead to further degradation and tool failure with reduced tool life. Polycrystalline diamond tools in turning zirconia have displayed adhesion wear mechanism with flank and cutting edge wear at maximum cutting speed of 400m/min, feed rate of 10 *µ*m/rev, and depth of cut 10*µ*m (50). Factors such as tool grit size, material hardness and its associated surface roughness have influenced the lifetime of dental CAD CAM milling tools [54].

The material response of ceramics as brittle and plastic deformation are often found to be influenced by cutting parameters such as feed rate, depth of cut and surface roughness. Removal mechanisms have been investigated in feldspathic, leucite and lithium disilicate glass ceramics [33,36,43]. Machined lithium disilicate glass ceramics have demonstrated distinct inter-granular and transgranular micro-fractures with brittle and plastic deformation [55]. Dental glass ceramics such as leucite and feldpathic porcelain under high speed dental hand-pieces with coarse diamond burs at a feed rate of 15-75 mm/min with a depth of cut of 10-60 *µ*m have demonstrated brittle fracture at high specific removal rate and ductile response at low specific removal rate [56]. Varied cutting parameters of simulated grinding on machined Y-TZP have resulted in micro-cracks, with rim damage of the substrate with plastic, mixed and brittle surface finish [57]. Hard turning of zirconia have resulted in surface roughness of Ra value below 0.6 µm, with lowest cutting parameters showed brittle-ductile removal mechanism [50].

The effect of high spindle speed of 16,000 rpm at a given depth of cut of 0.6 mm and feed rate 0.1 mm/tooth on zirconia reinforced mica glass ceramics can be clearly interpreted as brittle fracture response with micro-chipping as seen in Fig.3 and Fig.4. Grounding furrows on the ceramic substrate made by the machining tool can be seen in Fig.5. Studies on sub-surface damage in feldspathic porcelain and Dicor MGC glass ceramic revealed micro-cracking, chipping, crushing and crumbling. The response of Dicor MGC, a mica based glass ceramic, with CEREC-1 CAD CAM system and a high speed diamond wheel showed similar material removal mechanism of brittle fracture and micro-chipping to the experimental zirconia reinforced mica glass ceramic in the present study [58].

Limitations of the objective was the use of milling configuration of 16,000 rpm with a polycrystalline diamond tool insert against the high spindle speeds used in dental milling. As the study was on evaluating the feasibility of the new ceramic, the cutting parameters were based to avoid any possible damage to both the tool and the ceramic workpiece material. Further, investigations in a custom dental milling unit and its effect on diamond grit tool wear and material removal are recommended, for verifying its clinical application and hence the second objective of evaluating tool penetration rates was investigated.

### 4.2 Tool penetration rates and edge chipping

Essentially, the penetration depth of a material are directly related to the material removal rates and its machinability [27]. The greater penetration depth values of G20Z than its machinable counterpart, IPS e.max CAD (pre-crystalline lithium metasilicate) and PSZ (Pre-sintered zirconia) can be corroborated to the presence of fluorophlogopite plate like crystals in the microstructure of mica glass ceramics in G20Z and its relative ease of milling [59]. The optimum brittleness index of G20Z (2.5 *µ*m^-1/2^) with hardness of 9 GPa and fracture toughness of 3.6 MPa.m^1/2^ further supports the findings of the study [24]. Material removal rate depends on a number of factors such as tool grit size, tool design, tool sequence and the type of material, its surface hardness and surface roughness [60]. As the milling tools have been standardized in the present study, the variation in the tool penetration rates between the ceramic groups could be attributed to the differences in the mechanical properties of the investigated ceramics such as hardness, toughness and their related brittleness index [24,61,62]. The pre-crystallized IPS CAD with 40% lithium metasilicate interlocked platelet crystals of low aspect ratio in its glass matrix with brittleness index of 2.5~2.9 *µ*m^-1/2^ and hardness of 6~9 GPa is a well-known machinable ceramic. Lithium metasilicate in the pre-crystallized state offer sufficient strength for machining and requires an additional heat treatment for the precipitation of lithium disilicate crystals [63,64]. On the other hand, soft machining of pre-sintered zirconia (PSZ) though is time saving and wear effective, has limitations of fragility of pre-sintered ceramic bodies, with low dimensional tolerance and sintering treatment [65]. On the other hand, pre-sintered zirconia (PSZ), isostatically cold pressed at 11000C was reported with hardness of 6 GPa and fracture toughness of 0.7 MPa.m^1/2^, exhibits machinability, most influenced by the pre-sintering temperature (66,67). Further, soft machining of pre-sintered zirconia (PSZ) though is time saving, wear effective with reduced edge chipping, has limitations of fragility of pre-sintered ceramic bodies, with low dimensional tolerance [65].

Diamond particle wear on the cutting burs observed in experimental G20Z, commercially available zirconia (PSZ) and IPS e.max CAD, can be attributed to the high hardness of dental ceramics irrespective of their short milling time. The wear of the diamond tool in the all the investigated groups can be ascribed to the similar hardness of G20Z and IPS e.max CAD (~9 GPa).(24) Milling a hard ceramic and its consequent tool wear can shorten its life time, requiring frequent replacements of milling tool. In contrast, a soft dental material can chip and clog the milling tool as a residue, further reducing the cutting efficiency of the milling tool. Increased milling tool wear and missing diamond grains were reported with hard ceramic based CAD CAM material (VITA Mark II) and chip deposits on the milling tool were observed with soft polymer infiltrated composite CAD CAM blocks (VITA Enamic and Lava Ultimate) with Lyra conical tools (1 and 1.05 mm, GACD SASU) and CEREC cylinder pointed tools (12S) [54]. In contrast, absence of diamond pull-out or abrasion was observed on CAD CAM ceramics (IPS e.max CAD, Celtra Duo) and polymer infiltrated blocks (Lava Ultimate and Vita Enamic) with the milling tool (E4D Tapered 2016000; Premier) (4). In the extrapolation of the results of the experimental study to a typical CAD CAM dental milling set-up, the study has a few limitations of providing only vertical movement and using diamond burs due to the high cost in machining a new ceramic material in a typical CAD CAM system [4,27].

Edge chipping can be observed in all the ceramic specimens (Fig.4 (a-c)). The clinical relevance of marginal or edge chipping can be related to the required threshold of marginal gap (150 *µ*m) in indirect dental restorations and its associated risk of cement dissolution and secondary caries [68]. In a typical in-office CAD CAM milling set-up, the prosthesis or marginal fit is influenced by the embedded motor technology of the milling machines [27].

The porosities along the cutting surface of G20Z can be attributed to the pressure less sintering process followed for the heat treatment of G20Z. Irregular cutting surface of PSZ and smooth surface of IPS e.max CAD with edge chipping are likely due to the brittle nature of dental ceramics. Similar findings of margin chipping were reported in machinable ceramics such as IPS e.max CAD and Celtra Duo [4]. The smooth cutting surface of IPS e.max CAD can be endorsed to the manufacturing process and finishing of ceramic samples. Zirconia reinforced mica glass ceramics (G20Z) can be explored as alternate ceramic materials for dental restorations due to its chemical durability, innate machinable nature with optimum mechanical properties. Furthermore, advances in sintering procedures with high temperature and pressure can be explored for high densification of G20Z to prevent edge chipping for improved marginal fit.

## 5 Conclusion

From the results of the *in vitro* study, the following conclusions can be made:

1. The study was done to evaluate the feasibility of milling sintered 20 wt.% zirconia mica glass ceramic composites (G20Z). Thrust force, feed force and cutting force are influenced by spindle speed, depth of cut and feed rate. In particular, spindle speed and feed rate play a significant role in influencing surface roughness, thrust force, cutting forces and tool wear. High spindle speeds greater than 16000 rpm at a given depth of cut and feed rate for G20Z delivers low cutting forces with an average surface roughness of 1.61 *µ*m, resulting in abrasive wear of the tool insert and brittle fracture of zirconia mica glass ceramic composites.
2. G20Z with its machinable nature demonstrates greater tool penetration rates when compared with commercially available ceramics such as Pre-Sintered Zirconia and IPS e.max CAD at 30,000 rpm in a custom milling set-up.
3. Tool wear and edge chipping is seen in all the investigated ceramics. Advanced sintering techniques are recommended for G20Z to reduce edge chipping and tool wear.

## Figures and Tables

**Table I:** Experimental results of cutting forces and surface roughness of G20Z

Sl. No.	Spindle Speed (vc), rpm	Depth of cut (d), mm	Feed (f), mm/ tooth	Thrust force (Fz), N	Feed force (Ff), N	Cutting force (Fc), N	Surface Roughness (Ra), microns
1	8000	0.4	0.1	150	130	220	3.410
2	8000	0.6	0.2	168	289	240	3.970
3	8000	0.8	0.3	213	250	260	4.125
4	12000	0.4	0.2	128	84	110	2.625
5	12000	0.6	0.3	189	142	170	3.155
6	12000	0.8	0.1	170	166	138	3.380
7	16000	0.4	0.3	106	72	92	3.135
8	**16000**	**0.6**	**0.1**	**45**	**36**	**78**	**1.615**
9	16000	0.8	0.2	128	88	156	2.115

**Table II:** Analysis of Variance (ANOVA) results for Thrust, Feed, Cutting Force and Surface Roughness of G20Z

Parameters	Thrust force		Feed force		Cutting force		Surface roughness	
	F	p value	F	p value	F	p value	F	p value
**Spindle speed**	31.71	0.002*	20.46	0.006*	28.9152	0.003*	16.6621	0.009*
**Depth of cut**	11.51	0.019*	4.73	0.08	3.3467	0.12*	0.1567	0.70
**Feed rate**	6.96	0.046*	1.25	0.03*	1.2293	0.03*	3.1267	0.01*

**Table III:** Comparison of mean tool penetration rates between ceramic groups using one-way ANOVA Test

(I) Groups	(J) Groups	Mean Diff. (I-J)	95% CI for the Diff.		P-Value
			Lower Upper		
**IPS CAD**	PSZ	-0.0439	-0.1361	0.0483	0.48
	G20Z	-0.1100	-0.2044	-0.0155	0.02*
**PSZ**	G20Z	-0.0661	-0.1606	0.0284	0.21

**Table IV:** Multiple comparison of mean difference in tool penetration rates between the ceramic groups using Tukey’s Post hoc Analysis

Groups	N	Mean	SD	Min	Max	P-Value
**IPS CAD**	11	021	0.054	0.13	029	
**PSZ**	11	026	0.068	0.17	0.37	0.03*
**G20Z**	10	0.32	0.12	0.11	050	
**Groups**	**N**	**Mean**	**SD**	**Min**	**Max**	**P-Value**
**IPS CAD**	11	021	0.054	0.13	029	
**PSZ**	11	026	0.068	0.17	0.37	0.03*
**G20Z**	10	0.32	0.12	0.11	050	
